# Repeatability in measuring curvature in Peyronie’s disease

**DOI:** 10.1093/sexmed/qfaf105

**Published:** 2025-12-26

**Authors:** Majken H Wiborg, Rasmus Krøijer, Else B Kallestrup, Birgitte S Laursen, Gabriele Berg-Beckhoff, Lars Lund

**Affiliations:** Clinic of Sexology, Region of Southern Denmark, 6700 Esbjerg, Denmark; Faculty of Health Sciences, University of Southern Denmark, Region of Southern Denmark, 5000 Odense, Denmark; Faculty of Health Sciences, University of Southern Denmark, Region of Southern Denmark, 5000 Odense, Denmark; Department of Surgical Gastroenterology, Hospital South West Jutland, Region of Southern Denmark, 6700 Esbjerg, Denmark; Faculty of Health Sciences, University of Southern Denmark, Region of Southern Denmark, 5000 Odense, Denmark; Department of Urology, Hospital South West Jutland, Region of Southern Denmark, 6700 Esbjerg, Denmark; Sexology Centre, Aalborg University Hospital, Region of Northern Denmark, 9000 Aalborg, Denmark; Sexology Research Centre, Department of Clinical Medicine, Aalborg University, Region of Nothern Denmark, 9000 Aalborg, Denmark; Faculty of Health Sciences, University of Southern Denmark, Region of Southern Denmark, 5000 Odense, Denmark; Department of Regional Health Research, Hospital South West Jutland, Region of Southern Denmark, 6700 Esbjerg, Denmark; Faculty of Health Sciences, University of Southern Denmark, Region of Southern Denmark, 5000 Odense, Denmark; Department of Urology L, Odense University Hospital, Region of Southern Denmark, 5000 Odense, Denmark; Department of Urology, Aalborg University Hospital, Region of Nothern Denmark, 9000 Aalborg, Denmark; Faculty of Medicine, Aalborg University, Region of Northern Denmark, 9000 Aalborg, Denmark

**Keywords:** penile induration, reproducibility of results, observer variation, goniometer, photography

## Abstract

**Background:**

Despite guideline recommendations to assess Peyronie’s disease (PD) curvature during an intracavernous injection-induced erection, no standardized measurement protocol exists, and considerable interobserver variability remains.

**Aim:**

To evaluate the interobserver reliability of penile curvature measurements in patients with PD and to determine if image annotation enhances measurement consistency.

**Methods:**

In this study, 4 experienced urologists independently assessed erect penile curvature in 22 male patients enrolled in a prospective PD clinical trial. Two image sets per patient were analyzed: 1 consisting of self-captured photographs taken at home and the other obtained in the clinic after intracavernosal injection of alprostadil. Each image set included standardized lateral and dorsal views. The potential impact of an assisting line on photographic measurements was also evaluated.

**Outcomes:**

Interobserver agreement in curvature measurement, the effect of line annotation on measurement accuracy, and the comparison of measurement consistency between home-acquired and clinic-induced erection images.

**Results:**

Interobserver agreement was high, with Intraclass Correlation Coefficient values ranging from 0.77 to 0.88 across assessments. No statistically significant improvement in repeatability was observed with the addition of assisting lines. Furthermore, no meaningful difference in reproducibility was detected between home-based and pharmacologically induced image sets.

**Clinical Implications:**

Accurate measurement of penile curvature is crucial for comparing studies and evaluating treatment results. Variations in measurement techniques can lead to inconsistent data interpretation and reduce generalizability.

**Strengths and Limitations:**

This study highlights the need to standardize PD curvature assessment. While using multiple expert raters increases reliability, the small sample size and subjective interpretation of curvature may limit its broader use.

**Conclusion:**

Penile curvature measurements in PD show high interobserver reliability, regardless of photographic conditions or the use of assisting lines.

## Introduction

Peyronie’s disease (PD) is an acquired connective tissue disorder characterized by the formation of fibrotic plaques within the tunica albugínea (TA). These plaques lead to a localized loss of elasticity, resulting in the asymmetric expansion of the corpora cavernosa during erection, which ultimately causes penile curvature and often additional deformities, such as indentation or an hourglass appearance. The condition affects between 3.2% and 20.3% of men.^1–3^ It is associated with sexual dysfunction and psychological distress, resulting in a reduced quality of life.^4^ Accurate and reproducible assessment of penile curvature is crucial in clinical practice, as it is essential for informed decision-making, monitoring disease progression, and evaluating therapeutic trials.^5^

Although the American Urological Association (AUA) and European Association of Urology (EAU) guidelines recommend in-office, intracavernous injection (ICI)-induced erections as the standard for evaluating penile curvature before invasive treatment, they offer limited guidance on how to measure curvature in practice. Important procedural factors—such as the choice of measuring device, penis positioning, and how to handle situations where standard goniometers physically encounter the patient’s body—are not standardized. Consequently, clinicians and researchers depend on local practices, which leads to variability in both clinical assessments and study results.^6^^,^^7^

The goal of this study is not to question the guideline-supported use of ICI-induced erections but to assess the reproducibility of curvature measurements across common measurement methods and to highlight the need for more detailed methodological standardization to enhance comparability in clinical care and research.

Clinical and research consequences of inconsistent penile curvature measurements

- Clinical care variability: It is often difficult to determine whether a new PD treatment is truly effective when reported improvements of 10-20° fall within the known measurement error range. Such variability can obscure genuine therapeutic benefit or, conversely, suggest efficacy where none exists.^6^
- Research comparability: interobserver variability and methodological inconsistency hinder comparisons across studies, thereby limiting the validity of meta-analyses.^7^
- Under- or misreporting: Patient-generated photos often underestimate curvature compared with ICI-induced assessments, further reducing measurement reliability.^8^
  These methodological limitations emphasize the unmet need for reproducible, precise, and practical assessment modalities. Without standardized measurement protocols, both clinical decision-making and research conclusions are susceptible to bias, reduced precision, and limited generalizability.
  To address these gaps, we conducted a feasibility study exploring the reliability and reproducibility of multiple photographic measurement approaches in men with PD.
  We hypothesized that the curvature measurements would vary depending on:

1. The observer performing the assessment (inter-observer variation)
2. Whether the curvature was marked with assisting lines prior to measurement and,
3. Whether the photographs were taken by the patient at home or by a clinician in the office following ICI.

Does the use of annotated versus unannotated photographs, and home-produced versus clinic-produced photographs, affect the interobserver reliability of penile curvature measurements in men with PD?

## Materials and methods

### Materials

**Figure 1:**
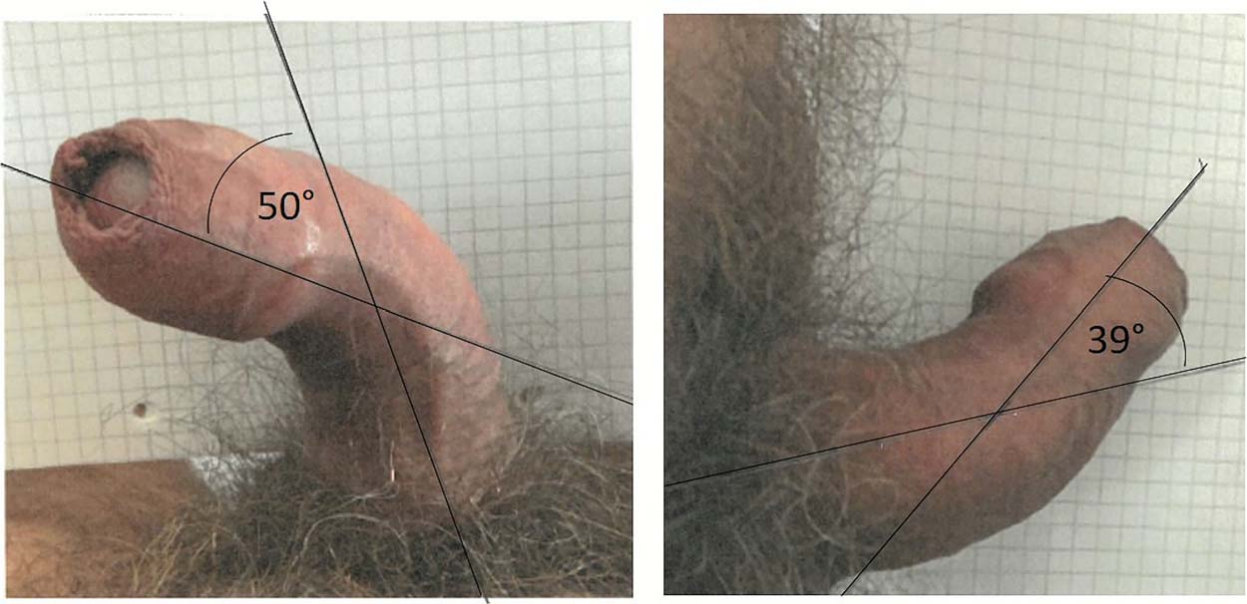
Penile picture of a participant. It shows how assisting lines were drawn.

This study included photographs of 22 Danish patients participating in a clinical trial.^9^ The inclusion and exclusion criteria are listed there. The patients were seen at the outpatient clinic, and a single investigator diagnosed PD based on the patient’s history and photographs of the erect penis (taken at home). The inclusion criteria for the trial were penile deviations between 30 and 90°. Each patient submitted 2 sets of pictures, each containing lateral and dorsal views of the erect penis (Figure 1). One set consisted of patient-produced photographs (PPP). The patients received a standard operating procedure for taking the PPPs at home. The instructions included 2 pictures of a penile model from lateral and dorsal views. Patients were instructed to use laminated checkered paper as the background and to place an adjustable constriction device around the base of their penis. This aimed to standardize the rigidity of the erection for the photographs.

The second set of photos included investigator-produced photos (IPP) taken in a clinical setting during a full pharmacologically induced erection with 10 mcg/mL alprostadil. To ensure a complete erection, dynamic Doppler ultrasound was performed at intervals of 5, 10, 20, and up to 30 minutes after alprostadil injection to monitor penile rigidity. If the patient did not achieve complete rigidity, additional alprostadil doses or self-stimulation privately were offered. All participants ultimately achieved a full erection, and no patients were excluded due to insufficient rigidity. All photos were taken by a single investigator.

### Methods

**Figure 2:**
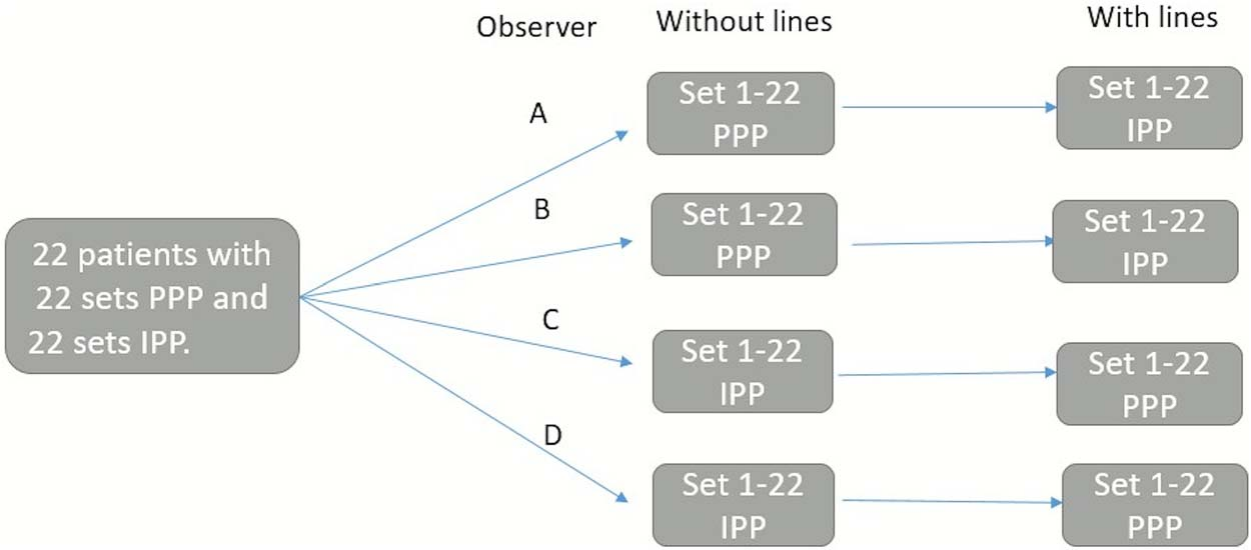
How the photos were distributed among the 4 urologists.

These photos were printed, anonymized, and provided to 4 experienced urologists. The group had an average age of 60 and an average of 18.75 years of experience. They had 16.25 years of experience in diagnosing and treating PD patients. Urologists 1 and 2 measured the lateral and dorsal curvature retrospectively and independently without assisting lines in the 22 PPPs, while Urologists 3 and 4 did so in the 22 IPPs without lines (Figure 2).

2 to 4 weeks later, urologists received another set of measures to prevent recall bias. Urologists 1 and 2 measured 22 IPPs, following instructions to draw two intersecting lines through the center of the distal and proximal penile shafts before taking measurements. Urologists 3 and 4 performed the exact measurements on the 22 PPPs (Figure 2), also using assisting lines. Each curvature was measured 3 times and recorded in a table. A translucent, plastic goniometer (ProTerapi A/S) was used. The PD assessment device developed by Dr. J. Mulhall was initially tested; however, reading an exact value was difficult due to the 5° spacing between lines.

### Statistical analyses

The 3 estimated measurements per urologist are summarized as means. We used an Intraclass Correlation Coefficient (ICC), a scatterplot, and a Bland–Altman plot to assess repeatability. The following cutoff points were used to evaluate ICC: values close to 1 indicated excellent agreement, and values between 0.75 and 0.90 indicated good agreement.^10^ Based on previous studies and clinical guidelines, a difference of 10° or more is often considered clinically meaningful in evaluating penile curvature.^8^^,^^11^^,^^12^ However, in this study, the limited sample size prevented reliable statistical testing of differences above or below this threshold. Stata version 18 was used for the statistical analysis.

### Ethical consideration

Given the photos’ delicacy, they were handled with respect and kept anonymous. The angle measurements were part of a clinical study investigating the effects of stromal vascular fraction cells on PD.^9^ Journal number 21/34757 and the National Committee on Health Research Ethics (74705) approved this investigation. The patient provided written informed consent to publish the photographs (Figure 1).

## Results

The mean age of the 22 men participating was 58.6 ± 9 years [44-74]. Analysis of penile curvature showed varied orientations: 45.6% dorsolateral (left), 36.1% dorsal, 13.7% left-lateral, and 4.6% ventral. The degrees of curvature were distributed as follows: 54.5% exhibited curvature of 30-60°, and 45.5% exceeded 60°; palpable plaques were present in all cases. When comparing the lateral and dorsal views, the mean measured curvature angles were higher in the IPP than in the PPP. In the lateral view, mean angles ranged from 50.16 to 61.22° in IPP, vs 41.47 to 47.17° in PPP. In the dorsal view, IPP ranged from 27.36 to 33.17°, while PPP ranged from 25.38 to 26.48°. Nonetheless, no difference was observed between images with and without lines (Tables 1 and 2).

**Table 1:** Measurements from urologists 1 and 2.

	**Patient-produced pictures (PPP) (without lines)**		**Investigator produced pictures (IPP) (with lines)**	
	**Physician 1**	**Physician 2**	**Physician 1**	**Physician 2**
**Lateral view**				
Mean	41.47	47.17	54.24	61.22
SD	14.76	16.90	19.51	27.72
ICC (95% CI)	0.77 (0.52-0.89)		0.54 (0.17-0.77)	
**Dorsal view**				
Mean	26.33	26.48	33.17	28.00
SD	20.51	18.63	25.37	25.81
ICC (95% CI)	0.92 (0.83-0.97)		0.71 (0.41-0.87)	
Missing’s	0	0	2	8

Abbreviation: ICC, Intraclass Correlation Coefficient.

**Table 2:** Measurements from urologists 3 and 4.

	**Investigator-produced pictures (IPP) (without lines)**		**Patient produced pictures (PPP) (with lines)**	
	Physician 3	Physician 4	Physician 3	Physician 4
**Lateral view**				
Mean	50.16	54.47	46.56	44.89
SD	21.99	20. 51	16.63	13.92
ICC (95% CI)	0.87 (0.72-0.94)		0.87 (0.71-0.94)	
**Dorsal view**				
Mean	32.15	27.36	25.38	26.39
SD	27.70	22.89	16.34	17.70
ICC (95% CI)	0.87 (0.66-0.95)		0.88 (0.73-0.96)	
Missing’s	7	1	4	0

Abbreviation: ICC, Intraclass Correlation Coefficient.

**Figure 3:**
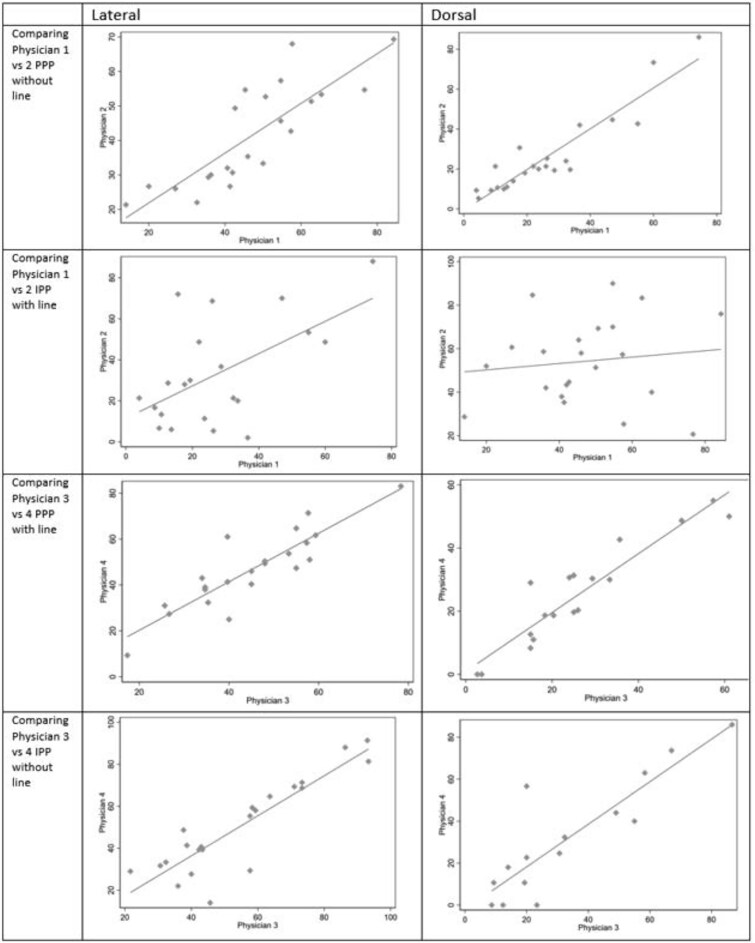
Scatter plots of measurements.

The scatter plots (Figure 3) show acceptable linear correlations between raters for PPP with assisting lines and IPP without lines for urologists 3 and 4, and for PPP without lines for urologists 1 and 2. In contrast, the scatter plots of urologists 1 and 2’s ratings of IPP with lines did not demonstrate a consistent linear pattern, as further supported by the Bland–Altman plots (Appendix 1).

The interobserver correlation coefficient was high, with ICCs ranging from 77 to 88 across most repeated measures. However, comparing urologists 1 and 2 for IPP and the line showed only moderate repeatability (ICC = 0.54) (Table 1). Compared with IPPs, PPPs underestimated the lateral and dorsal view curvatures, although dorsal view measurements ranged from 25.38 to 33.17° and showed only minor differences.

## Discussion

### Principal findings

This study revealed notable differences among observers in measuring penile curvature, regardless of whether assisting lines were used. Although the overall interobserver correlation was high (ICC > 0.75), specific discrepancies—particularly between urologists 1 and 2—were observed when assessing IPP with lines. This indicates that, while reproducibility is generally acceptable, variability remains a concern in more complex clinical photographs. Importantly, PPP showed the **least variability,** possibly because the curvatures were less severe than those after ICI. Unexpectedly, adding assisting lines did **not** improve measurement reproducibility.

Similarly, we found no consistent advantage of PPP over ICI photographs. Interestingly, PPP displayed less variability among raters, which might be due to the simpler curvature in these images and the inherent challenges of post-ICI photography, such as varying erection rigidity and degrees of curvature severity.

Notably, the present study assesses *reliability* rather than *accuracy*, as no gold-standard live goniometric ICI measurements were included for direct comparison. Our findings, therefore, provide preliminary evidence that commonly used photographic methods can achieve reasonable interobserver consistency but should not be taken as proof of measurement validity. Nonetheless, standardized photographic protocols may have significant value for research purposes and for remote or long-term follow-up, especially as digital tools improve. Emerging app-based assessment technologies and early studies using artificial intelligence to measure penile curvature further highlight the potential for automated, reproducible, and patient-friendly measurement techniques. The present study offers a foundation for these advancements and underscores the need for future validation against the in-office reference standard.

### Comparison with previous literature

Our findings align with previous research emphasizing the challenge of objectively measuring penile curvature in PD. A 2022 study^13^ that used a plastic 3D model of PD showed similar variability among providers, highlighting the need for better standardized tools. Other studies consistently reveal differences between patient-reported and clinician-assessed curvatures. For example, in a 2014 study,^14^ nearly half of the patients misjudged their curvature compared to assessments based on ICI. Similarly, drawing-based methods indicated that patients often overestimate their curvature, supporting our conclusion that patient estimates cannot replace standardized evaluations.^15^ Although ICI remains the gold standard for inducing erections during diagnostic assessments, inconsistent image capture and measurement techniques limit reproducibility. Ohebshalom et al.^16^ and Traeger et al.^8^ both found that ICI tends to expose more severe deformities than AHP, which is consistent with our findings. However, many of these studies lack essential details about measurement methods, which hampers cross-study comparisons.

### Guideline recommendations lack standardization in measurement

Although major urological societies—including the AUA**,** Canadian Urological Association (CUA), and the EAU—recommend in-office, ICI-induced erection as the standard test for assessing penile curvature, their guidelines lack detailed, standardized protocols on how to perform curvature measurements. For instance, the AUA guideline (2015) endorses ICI testing (with or without Doppler) for evaluating deformity, length, and curvature, but does not specify the measurement device, patient positioning, or photographic technique.^17^ Similarly, the 2018 CUA guideline recognizes post-ICI examination as the “gold standard” and permits measurement with a protractor on full-erection digital photographs, but omits detailed step-by-step instructions.^18^ Lastly, although the EAU also supports assessing curvature in the erect state, its publicly available guidance does not recommend a consistent method for angle measurement, leading to ongoing variability in practice.^19^ This lack of detailed procedural instructions leads to significant differences in measurement methods (eg, goniometer choice, patient positioning, angle photography), likely contributing to interobserver differences of up to 10-20° reported in clinical and research settings.^6^ Such variability can hinder distinguishing actual therapeutic effects from measurement noise in interventional studies.

### Reflection of the Kelâmi method

In 1983, Kelâmi^20^ introduced a method involving 5 reference lines drawn on self-taken penile photographs (known as “auto photographs”) to improve measurement accuracy. While innovative at the time, this approach has significant limitations that hinder its widespread clinical use. The method lacks formal validation, especially without standardized training. Additionally, Kelâmi’s technique does not account for complex 3D deformities, such as hinge effects or hourglass narrowing, which are essential in modern clinical assessment. Although Kelâmi’s classification system, including objective criteria (induration, size, localization, deviation, and number of plaques) and two subjective parameters (pain and difficulty with penetration), remains influential, his proposed measurement method is no longer sufficient for reliable curvature evaluation. Our findings further emphasize that adding lines—even in a simplified form—did not improve interobserver agreement, underscoring the need for a more standardized and validated approach.

Reviews by Levine and Greenfield^11^ and Müller and Mulhall^12^ continue to highlight the urgent need for a standardized methodology, especially regarding erection induction, image quality, and evaluator consistency. The authors of this article found that only 61% of the articles reported by Müller and Mulhall in their paper^12^ used picture-based curvature assessment, and only 15% of these articles explained how the measurements were done.^21–33^ Ziegelmann et al. also pointed out this lack of methodological transparency in 2020.^6^

### Clinical and research implications

Despite its limitations, PPP can still serve as a useful initial, less invasive method to assess penile curvature, particularly in patients hesitant to undergo ICI. However, variability among raters, especially with complex IPP images, indicates that photographic methods alone are not yet dependable enough to replace in-office evaluations. Our results emphasize the importance of detailed method reporting in future studies—such as how erections are induced, how photos are taken, and how curvature is measured (for example, using a goniometer versus digital tools). Until validated alternatives are available, the gold standard should remain an assessment by a provider, involving direct angle measurement with a protractor or goniometer and comprehensive documentation of deformity, including curvature degree, laterality, rigidity, narrowing, and hinge effects. Emerging tools like 3D scanning, structured light, and AI-supported smartphone apps might eventually offer more standardized, user-friendly options, but they require thorough validation.

In addition to interobserver reliability, intraobserver consistency is another crucial dimension that deserves attention. Because clinical decision-making often depends on detecting relatively small changes in curvature over time, future studies would benefit from evaluating how consistently the same observer measures identical images across different time points.

### Strengths

This study addresses a crucial clinical gap in evaluating the reproducibility of various photographic methods for measuring penile curvature in PD. The findings suggest that, under certain conditions, the PPP may provide useful estimates of curvature in PD patients, thereby streamlining clinical assessments.

### Limitations

A small sample size and moderate statistical power limited the feasibility of this study. Variability in image quality, especially in IPP, along with the complexity of deformities such as torsion and multi-plane curvature, could have affected measurement accuracy. Some urologists found certain images too difficult to assess reliably, leading to missing data. Although instructions were standardized, interpretation probably varied among observers. Additionally, the PPP method may be less practical for patients with ED or those who have difficulty capturing photos from the correct angle.

This study did not conduct live goniometric measurements during ICI-induced erections, which limits our ability to directly compare photographic measurements with the guideline-recommended reference standard.

We used a mild constriction device to standardize erection rigidity, but this approach is not part of standard curvature assessment. We cannot fully rule out that it may have influenced the curvature’s appearance.

## Conclusion

Our feasibility study revealed significant variability among observers in measuring penile curvature, and the addition of assisting lines did not improve reproducibility. No consistent differences were observed between measurements on PPP and those taken after ICI. Therefore, the findings mainly reflect the reliability of these commonly used methods rather than their accuracy.

Although the small sample size limits definitive conclusions about the best measurement technique, the results highlight ongoing methodological challenges in assessing curvature. Standardized photographic protocols could improve reproducibility in both research and remote follow-up, especially with new app-based and AI-assisted tools for measuring penile curvature.

This study should therefore be seen as an initial assessment of reproducibility that sets the stage for future validation research. These upcoming studies will directly compare photographic measurements with the gold standard, in-office live goniometric evaluations recommended by current guidelines. Consistent, step-by-step measurement protocols based on consensus remain essential for improving comparability across clinical practice and research.

## Supplementary Material

Appendix_1_1_qfaf105
