# Supplementary figures and images for: Repeatability in measuring curvature in Peyronie’s disease
Source: Sex Med. 2025 Dec 26;13(6):qfaf105. doi: 10.1093/sexmed/qfaf105 (PMC12728819; doi:10.1093/sexmed/qfaf105)

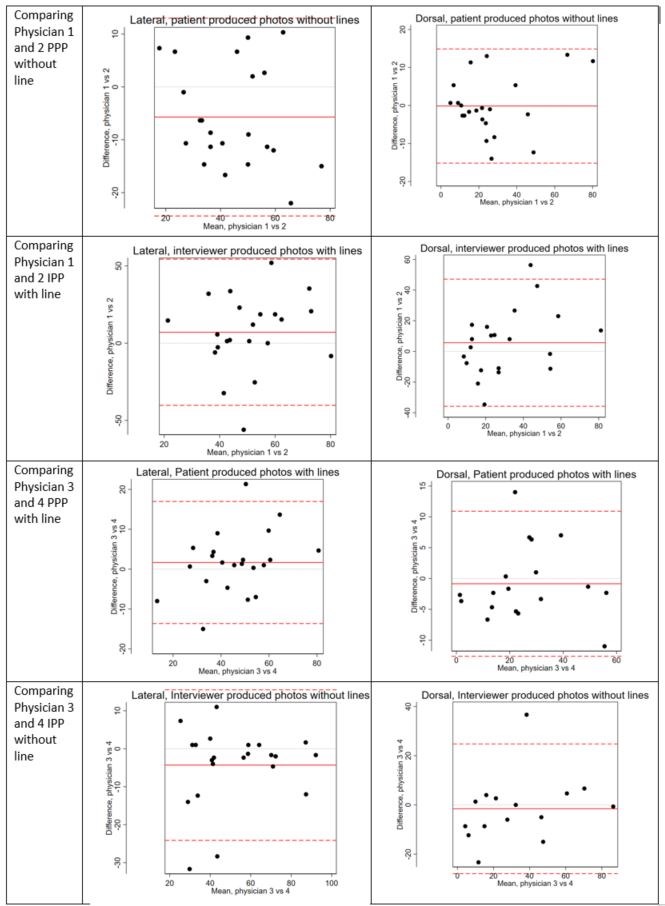

Supplement: Appendix_1_1_qfaf105 [file appendix_1_1_qfaf105.jpeg]
